# Gene Expression Patterns during the Early Stages of Chemically Induced Larval Metamorphosis and Settlement of the Coral *Acropora millepora*

**DOI:** 10.1371/journal.pone.0091082

**Published:** 2014-03-14

**Authors:** Nachshon Siboni, David Abrego, Cherie A. Motti, Jan Tebben, Tilmann Harder

**Affiliations:** 1 Australian Institute of Marine Science, Townsville, Australia; 2 School of Biological, Earth and Environmental Sciences, Centre for Marine Bio-Innovation, The University of New South Wales, Sydney, Australia; Laboratoire Arago, France

## Abstract

The morphogenetic transition of motile coral larvae into sessile primary polyps is triggered and genetically programmed upon exposure to environmental biomaterials, such as crustose coralline algae (CCA) and bacterial biofilms. Although the specific chemical cues that trigger coral larval morphogenesis are poorly understood there is much more information available on the genes that play a role in this early life phase. Putative chemical cues from natural biomaterials yielded defined chemical samples that triggered different morphogenetic outcomes: an extract derived from a CCA-associated *Pseudoalteromonas* bacterium that induced metamorphosis, characterized by non-attached metamorphosed juveniles; and two fractions of the CCA *Hydrolithon onkodes* (Heydrich) that induced settlement, characterized by attached metamorphosed juveniles. In an effort to distinguish the genes involved in these two morphogenetic transitions, competent larvae of the coral *Acropora millepora* were exposed to these predictable cues and the expression profiles of 47 coral genes of interest (GOI) were investigated after only 1 hour of exposure using multiplex RT–qPCR. Thirty-two GOI were differentially expressed, indicating a putative role during the early regulation of morphogenesis. The most striking differences were observed for immunity-related genes, hypothesized to be involved in cell recognition and adhesion, and for fluorescent protein genes. Principal component analysis of gene expression profiles resulted in separation between the different morphogenetic cues and exposure times, and not only identified those genes involved in the early response but also those which influenced downstream biological changes leading to larval metamorphosis or settlement.

## Introduction

Among the cues that trigger the pelago-benthic transition of coral larvae, biomolecular cues associated with the natural habitat, such as coral rubble, crustose coralline algae (CCA) and marine bacteria, have received considerable attention in the literature [1]–[4]. Previous investigations of gene expression profiles in coral larvae after exposure to these natural morphogenetic cues have focussed on the regulation of candidate genes with presumptive key functions in coral development, such as those implicated in cell proliferation, apoptosis, differentiation, migration, adhesion, biomineralization and immunity [5]–[11]. These investigations identified a large number of genes that were differentially expressed across the complete developmental transition, from competent swimming larvae to post-metamorphosis primary polyps [7], and that regulatory changes in gene expression occurred between 4 to 12 h post incubation (hpi) to a variety of morphogenetic stimuli [6]–[8], [12]. The general conclusion was that, at the transcriptional level, coral larvae appear to anticipate metamorphosis [6].

Changes in gene expression profiles of larvae exposed to morphogenetic stimuli have mostly been investigated using non-targeted approaches [6], [8] primarily due to the variable and complex composition of coral rubble obtained in the field, typically characterized by a mix of live and dead CCA, endolithic green algae, polychaetes, diatoms, protozoa and bacteria. Further compounding this issue is the likelihood that changes to the gene expression profiles of larvae exposed to CCA are in fact an integrated response to a multitude of qualitatively different triggers possibly affecting gene expression at multiple and unrelated levels.

A bioassay-guided chemical isolation strategy was employed to generate chemically refined and quantifiable putative morphogenetic cues from these natural biomaterials that would trigger larval morphogenesis *in vitro*. This strategy yielded (1) an organic and an aqueous fraction of the CCA *Hydrolithon onkodes* (Heydrich) that, under laboratory conditions, triggered in a highly reproducible manner, settlement, characterized by attached metamorphosed juveniles [13] and (2) a crude extract of *Pseudoalteromonas* strain J010 (J010-E) isolated from *H. onkodes*, known to contain tetrabromopyrrole (TBP), that triggered larval metamorphosis, characterized by non-attached metamorphosed juveniles [4], [12].

The conceptual approach taken in the present study targeted genes of interest (GOI) putatively involved in the early stages of coral larval morphogenesis (1–3 hpi), using multiplex RT-qPCR [12] to measure the gene response after short-term exposure to the chemically refined morphogenetic cues, and ultimately to discriminate between their possible modes of action. This approach had several advantages over previous studies: (1) it reduced the variance within biological replicates [14], (2) improved the resolution of gene expression levels, (3) lowered possible interferences from other components normally present in environmental biomaterials (see above) that may elicit non-specific changes in gene expression levels [7], and (4) decreased secondary time-related effects of cues on downstream gene expression. Discussed here are the putative roles of GOI in the early stages of coral larval settlement and metamorphosis based on changes in their expression profiles prior to any observed morphogenetic transformation.

## Materials and Methods

### Ethics Statement

This work was undertaken under permit G10/33440.1, issued by the Great Barrier Reef Marine Park Authority. No ethical approval was required for any of the experimental research described herein.

### Sample collections and larval maintenance

Colonies of the scleractinian coral *A. millepora* were collected from Pelorus Island on the Great Barrier Reef (GBR; 18°33′S 146°29′E) and transported to the Australian Institute of Marine Science (AIMS) prior to the predicted spawning events in November 2010 and November 2011. Chips of the CCA *Hydrolithon onkodes* (Heydrich) were collected from Trunk Reef (GBR; 18°24′S, 146°48′E) and transported to AIMS in November of 2010, 2011 and 2012. The taxonomic identity of all CCA samples was confirmed prior to use [15]. Each CCA chip was carefully removed from the solid substrate using sterile bone cutters creating a thin layer without any other visible algae or invertebrates and subsequently washed with 0.2 µm filtered seawater (FSW). Coral larvae were obtained after fertilization of gametes as described previously [4]. Larvae were raised in flow-through 500 L tanks containing FSW at 27–28°C until they became fully competent, as characterised by at least 80% of larvae displaying an elongated shape, exhibiting active benthic searching behaviour and settlement upon exposure to CCA. In this controlled environment larvae are deemed competent approximately four days after fertilisation and can remain in this state for an additional ten days [12]. Their high lipid content is used for buoyancy and as an energy source [16] allowing them to survive at least three weeks after settlement without feeding.

### Growth conditions and extraction of metamorphosis-inducing bacteria

The larval metamorphosis-inducing cue was isolated from *Pseudolalteromonas* strain J010 (JF309049) [4]. Briefly, bacterial colonies (3.2 g) were carefully scraped off agar plates, suspended in 50 mL ethanol (64 mg/mL) and sonicated for 20 min (Soniclean 500T, SA). The resulting bacterial extract (J010-E) was filtered (0.2 µm) and stored in the dark at −20°C.

### Preparation of CCA-derived settlement cues

Two settlement-inducing cues, derived from an organic extract and an aqueous extract, were isolated from *H. onkodes.* An ethanol extract of *H. onkodes* (2300 g wet weight, November 2011) was fractionated on reversed phase C-18 vacuum flash chromatography (RP-18 VFC, 80×600 mm) by elution with Milli-Q water (MQW, 600 mL), 10% methanol in MQW (600 mL), 20% methanol in MQW (600 mL), 80% methanol (600 mL) in MQW and 100% methanol (1200 mL). The 80% methanol fraction (0.95 mg/mL; herein referred to as HO_org_) induced high rates of larval settlement in preliminary bioassays and was stored at −20°C [13].

The aqueous extract was prepared as follows: *H. onkodes* (500 g wet weight, November 2011) was washed with 500 mL MQW three times to remove salts. These washings were discarded. The washed CCA were autoclaved at 121°C for 60 min in 250 mL MQW. This aqueous extract was decanted and the algae autoclaved twice more (121°C for 60 min in 250 mL). The pooled extract was ultrafiltrated (Grace Microcon-100, >100 kD), freeze-dried, resuspended in 250 mL 10% ethanol (56.1 mg/mL) in MQW, herein referred to as HO_aq_, and stored in the dark at 4°C [13].

HO_org_ was extracted over three consecutive years (2010–2012); HO_aq_ was extracted over two consecutive years (2011–2012). Comparison of the proton nuclear magnetic resonance spectra of each of these samples confirmed their chemical composition was consistent (data not shown).

### Determination of minimum exposure time of larvae to cues that elicit settlement and/or metamorphosis

Preliminary experiments were conducted using 6–9 day-old swimming larvae in 6-well plates. In 2010, larvae were exposed to J010-E (Table 1) to assess the minimum required exposure time of larvae to the metamorphosis-inducing cue. The extract J010-E (2 µL; 128 µg/mL) or 100% ethanol (2 µL negative control) were added to 10 mL of 0.2 µm FSW, mixed thoroughly and transferred to 6-well plates (Nunclon Delta Surface, Thermo Scientific, Denmark; final concentration 12.8 µg/mL). Larvae were added to each well (n = 20 per well) and monitored hourly for visible signs of metamorphosis without settlement, characterised by flattening into discs and septal development in floating polyps. Following the same protocol, swimming larvae were exposed to settlement-inducing cues HO_org_ (evaporated on the dish surface before adding FSW) or HO_aq_ (aliquot added to FSW) in 2011. No cue was added to the control treatments (n = 6). In a preliminary experiment, addition to the well and evaporation of the carrier solvents (10% ethanol, 100% ethanol, 80% methanol and MQW) to dryness resulted in no settlement, as observed with the no solvent (blank) control (A. Negri, personal communication), and therefore only the blank control was used.

**Table 1:** Experimental parameters.

Cue	HO_org_	HO_aq_	J010-E
**Origin of Cue**	*Hydrolithon* onkodes (Heydrich) crustose coralline alga (CCA)		Biofilm of *Pseudoalteromonas* strain J010 (JF309049)
**Larval response**	Settlement & metamorphosis		Metamorphosis without attachment
**Spawning Year**	2011		2010
**Larval age**	6 days		9 days
**Larval numbers**	100–200		200–300
**Treatment volume**	100 mL		300 mL
**Exposure time**	1 hpi		1–3 hpi
**Replicates**	6		18 (6 per h)
**Aliquot (µl)**	25 µL		60 µL
**Application**	Dry	FSW	ethanol
**Final cue concentration**	238 ng/mL	14 µg/mL	12.8 µg/mL
**Settlement/metamorphosis rate**	16 hpi 94±5%	16 hpi 70±8%	6 hpi 98±2%
**Assay 1 – Most stable genes**	*Rps7*, *ATF4/5*		*Rpl9*, *CTL-2*
**Average %CV ± STDEV (assay 1)**	6.9±5.1		8.2±5.5
**Assay 2 – Most stable genes**	*Rps7, Amgalaxin-like-1*		*amilRFP*, *Rps7*
**Average %CV ± STDEV (assay 2)**	9.6±5.8		7.7±6.5

### Experimental design and sample collection

Gene expression profiles may vary considerably throughout the different coral life phases; pre-settlement motile larvae to post-settlement metamorphosed primary polyps [7]. To avoid any variation due to differences in larval development and to accomplish meaningful comparisons between larvae exposed to control conditions versus J010-E or CCA-derived HO_org_ and HO_aq_ cues, all assays were terminated before the onset of visible metamorphosis without attachment or settlement (as determined above). Assays with J010-E were terminated 1, 2 and 3 hours post incubation (hpi), whereas assays with HO_org_ and HO_aq_ were terminated after 1 hpi (see experimental parameters in Table 1).

The extract J010-E (60 µL; 3.84 mg/mL) or 100% ethanol (60 µL, control) was mixed with 250 mL FSW in 400 mL glass beakers (Boeco, Germany; 6 replicates per treatment) to ensure homogeneous distribution prior to the addition of a further 50 mL FSW containing competent swimming larvae (six days-old, n∼300, final concentration 12.8 µg/mL). Aliquots of HO_org_ (25 µL; 23.8 µg/mL) were applied to the base of 400 mL glass beakers and allowed to evaporate, while HO_aq_ (25 µL; 1.4 mg/mL) was applied directly into the seawater (6 replicates per treatment). FSW (50 mL) was added to 400 mL glass beakers and gently mixed prior to the addition of 50 mL of FSW containing swimming larvae (nine days-old, n∼200) to give a final volume of 100 mL (final concentration HO_org_ 238 ng/mL and HO_aq_ 14 µg/mL). Blank treatments (no cues or solvents) were used as the negative controls.

In all experiments, an additional glass beaker containing each of the cues was left for 24 hpi to monitor larval metamorphosis and mortality. After each observation time point, larvae were collected on 5 µm, 25 mm diameter sterile filters under vacuum. Retained larvae were transferred into 1.5 mL cryovials, snap-frozen in liquid nitrogen and stored at −80°C.

### Multiplex RT-qPCR Assays

Forty-seven genes of interest (GOI) were selected for their putative role(s) in larval settlement or in coral immune defence pathways, and incorporated in two multiplex RT-qPCR assays (Assay 1; immunity-related genes potentially involved in ontogenesis, and Assay 2; genes reportedly involved in settlement; kanamycin (*Kan* ^r^) internal control gene in both assays) as previously described (Tables S1 and S2 in [12]). The suggested roles of the GOI in relation to marine invertebrate larval settlement and metamorphosis have been summarised and reported previously (Table 1 in [12]). One negative template (T−) and one negative Reverse Transcriptase (RT−; *Kan^r^* only) control was used in each assay to test for cDNA and RNA contaminants, respectively, according to the GenomeLab™ GeXP manual (a detailed description for this procedure is reported in [17]). For technical reasons the number of genes in every multiplex assay is limited to approximately 30 [12], [17]. The protocols for extraction of mRNA, preparation of cDNA, amplification by PCR and subsequent electrophoresis were adopted from Siboni et al. [12] with modifications: 1) larval mRNA was measured twice (NanoDrop ND-1000, Thermo Scientific, USA), averaged and diluted to 5 ng/µL (according to the GenomeLab™ GeXP manual); 2) 10 ng of mRNA was used in the reverse transcription mix; and 3) dilution of PCR products was changed to 1∶40 for J010-E and 1∶20 for HO cues. Diluted PCR products were analysed individually on an automated capillary electrophoresis sequencer (CEQ™ 8800 Genetic Analysis System, Beckman-Coulter, Fullerton, CA, USA) as follows: diluted PCR products (1 mL) were loaded into a 96-well plate with 0.2 µL of DNA size standard (400 bp; Beckman-Coulter) and 38.8 µL of Genome-Lab Sample loading solution. Electropherograms were visualized, filtered and matrices generated for further analyses following the GenomeLab™ GeXP manual (a detailed description for this procedure is reported in Souter et al. [17]).

### Statistical Analysis

Data were normalised against *Kan* ^r^. An expression stability measure for all genes in both assays was performed using the GeNorm program (http://medgen.ugent.be/genorm/) according to Vandesompele et al. [18]. The most stable pair of genes was used to normalize the gene expression levels of the GOI in Microsoft Excel (2007) using the Geomean function (Table 1). Further interrogation of the data was undertaken by normalisation using three of the most stable genes in every assay (Figure S1) and results compared with those from the pair of stable genes. For data analysis, only samples with at least two technical repeats and coefficient of variation percentage (%CV) lower than 25% were considered. Statistical analyses were conducted using Kruskal–Wallis (non-parametric one way analysis of variance by ranks) followed by multiple comparisons of mean ranks for all groups with STATISTICA version 10.0 (StatSoft, Tulsa, OK). Percentage differences of those genes that were differentially regulated from control were calculated. Further, data were analysed by principal component analysis (PCA) with PAST [19]. All PCA's were performed on a variance-covariance matrix, as all variables were measured in the same units, and without any group assumptions. PCA score plots are presented with convex hulls highlighting groupings and showing PC1 and PC2.

## Results

### Determination of minimum exposure times of larvae to cues to elicit settlement or metamorphosis without attachment

The minimum exposure time to elicit larval responses varied among the different cues. Visible signs of early metamorphosis (flattening into discs and development forming floating polyps) occurred 4 hpi with J010-E, whereas complete metamorphosis without attachment of 98±2% of larvae occurred 6 hpi. In order to sample swimming larvae prior to any observable morphogenetic changes, they were collected 1-3 hpi with J010-E (Table 1).

While no behavioural or developmental response was recorded during the first 2 hpi with HO_org_ and HO_aq_, initial attachment was observed after 3 hpi, and complete metamorphosis with attachment was observed 16 hpi (settlement rates of 94±5% (HO_org_) and 70±8% (HO_aq_), respectively). To reduce any variation in the assays and to ensure that all larvae were sampled at the same developmental phase, larvae were collected 1 hpi with HO_org_ or HO_aq_ (Table 1). It was noted that addition of the CCA-derived cues directly to larvae in FSW resulted in ∼5% mortality, as determined by the release of mucus resulting from tissue breakdown. This method of application was deemed unsuitable for the current study. Pre-conditioning the FSW with the CCA-derived cues followed by addition of larvae did not lead to any mortality (as determined above by release of mucus); therefore these conditions were used in the main experiment.

The larvae exposed to the three different cues displayed normal swimming behaviour with no morphological signs of settlement at the time of termination of experiments (1–3 hpi). With respect to the two CCA-derived cues, no larval response was recorded during the first 2 hpi. Furthermore, a subset of larvae settled, deposited a calcareous skeleton, divided and underwent complete metamorphosis. These juveniles were successfully infected with *Symbiodinium* (data not shown). No mucus resulting from tissue breakdown was observed 24 hpi in response to any of the three cues.

### Gene regulation/expression

**Figure 1:**
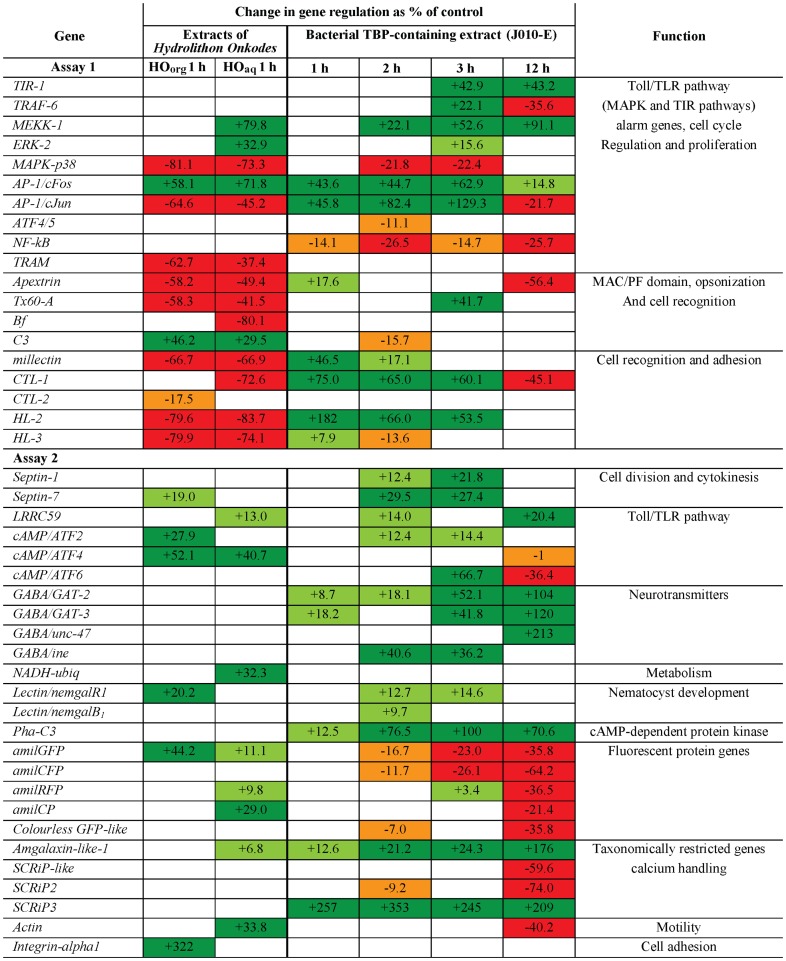
Differential gene expression following exposure to bacteria- and CCA-derived cues. Significant (*p<0.05*) change in gene regulation is given as the % difference compared to the control. Dark and light green (+) represents those genes that were up-regulated (X>20, 20≤X<0); orange and red (−) represents those that were down-regulated (0>X≥−20, X<−20). Data from the 12 hpi experiment (high concentration treatment and complete metamorphosis) was taken from Siboni et al. [12], which also includes full protein names and description of the genes.

Gene pairs used to normalize expression levels and their coefficients of variation (%CV) are listed in Table 1. For 11% of the total number of data points in this study two technical replicates were used instead of three, the third being unreliable. The negative template (T−) control did not produce any measureable products, as described by the GenomeLab™ GeXP manual, while a clear measureable peak at 325 nucleotide size was observed for the negative reverse transcriptase control (RT−; *Kan^r^* only), confirming that electropherograms resulting from the larval samples reflected transcribed gene amplification products. Only genes that changed significantly (*p<0.05*, Kruskal–Wallis followed by multiple comparisons of mean ranks) as compared to the control were further analysed (Figures 1 and S1). Two boxplot figures showing the variances, one for the CCA-derived cues, HO_org_ and HO_aq_ (Figure S2) and the other for the bacterial cue J010-E (Figure S3), represent the complete data. The averaged data is presented in Figure 1. The inclusion of a third gene in the normalisation had only a minor impact on the results (Figure S1), therefore only the two most stable genes, were used (Figure 1). It should be noted that the original reference genes included in the two assays [12] were not always the most stable genes according to GeNorm program [18] (Table 1). In all instances at least one of these stable genes was a ribosomal protein gene. Given that significant differences in gene expression were observed at the early time point and that no mucus strands, indicative of tissue breakdown, were observed 24 hpi with any of the cues, the concentrations used in this study were considered appropriate to measure gene expression levels while maintaining larval competency.

### Mutiplex RT-qPCR Assay 1; putative settlement/metamorphosis-related immunity genes

**Figure 2:**
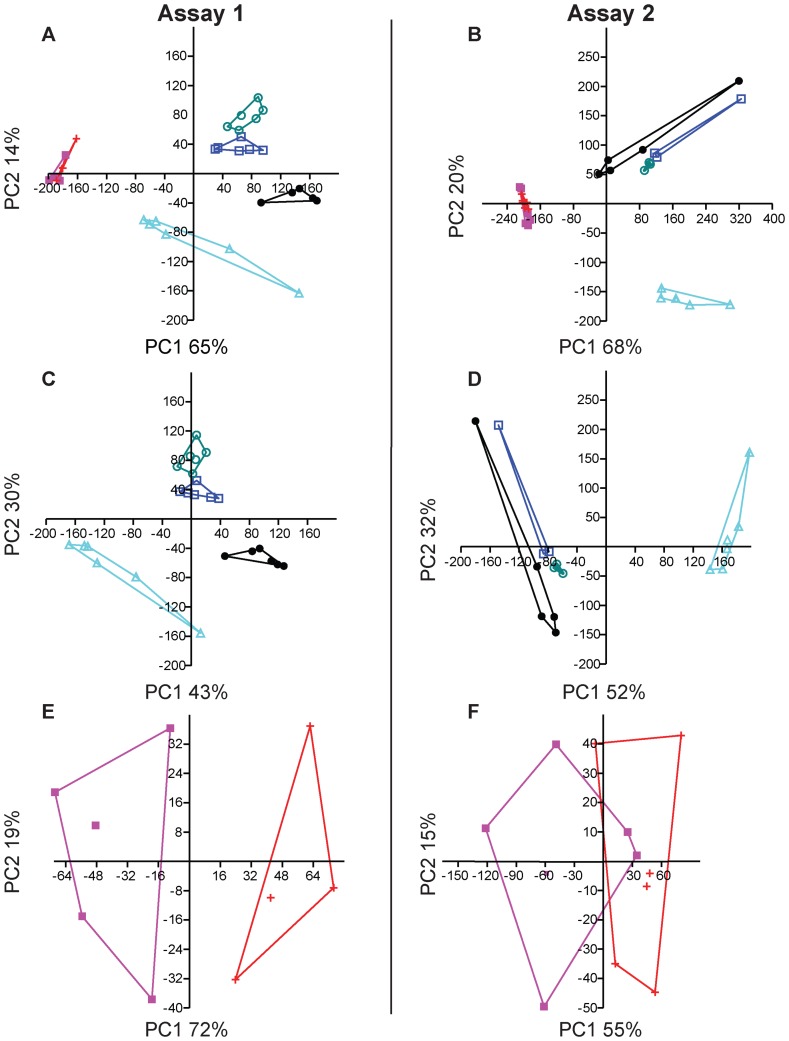
Influence of different settlement-inducing cues and exposure times on gene expression of *Acropora millepora* larvae. PCA score plots of the % difference in gene expression compared to the control, presented with convex hulls highlighting groupings and showing PC1 and PC2, for A) J010-E 1–12 hpi and HO_org/aq_, Assay 1, B) J010-E 1–12 hpi and HO_org/aq_, Assay 2, C) J010-E 1–12 hpi, Assay 1, D) J010-E 1–12 hpi, Assay 2, E) HO_org/aq_, Assay 1 and F) HO_org/aq_, Assay 2. Time points for J010-E are represented by: 1 hpi = black full circle, 2 hpi = blue empty square, 3 hpi = green empty circle and 12 hpi = cyan empty triangle. CCA-derived cues are represented by: HO_org_ = pink full square and HO_aq_ = red cross.

Expression levels of 16 GOI (*MEKK-1*, *ERK-2*, *MAPK-p38*, *AP-1/cFos*, *AP-1/cJun*, *NF-κB*, *TRAM*, *Apextrin*, *Tx60-A*, *Bf*, *C3*, *millectin*, *CTL-1*, *CTL-2*, *HL-2* and *HL-3*; Figure 1) changed significantly 1 hpi. During this time interval the lectins *millectin*, *CTL-1*, *CTL-2*, *HL-2* and *HL-3* were predominately up-regulated in response to the metamorphosis-inducing cue J010-E, whereas their down-regulation was observed in response to one or both of the settlement-inducing CCA-derived cues HO_org_ and HO_aq_. *AP-1/cFos* was consistently up-regulated in response to J010-E, HO_org_ and HO_aq_. The genes *TIR-1*, *TRAF-6*, *MEKK-1*, *ERK-2*, *MAPK-p38*, *ATF4/5*, *Tx60-A* and *C3* (Figure 1) caused significant differences 2 and/or 3 hpi with J010-E, whereas *AP-1/cFos*, *AP-1/cJun*, *NF-κB*, *CTL-1* and *HL-2* were significantly regulated at all three sampling time points (Figure 1). Some genes were similarly regulated following exposure to HO_org_ and HO_aq_ (*MAPK-p38*, *AP-1/cFos*, *AP-1/cJun*, *TRAM*, *Apextrin*, *Tx60-A*, *C3*, *millectin*, *HL-2* and *HL-3*) while others were differentially regulated (as compared to control) after exposure to only one of the HO cues (*MEKK-1*, *ERK-2*, *Bf*, *CTL-1* and *CTL-2*; Figure 1). The PCA score plot (Figure 2A) explained 79% of the variance and revealed clear differences in gene regulation following exposure to J010-E or the CCA-derived cues. Further PCA considering only the different exposure times to J010-E (Figure 2C) and another comparing the organic and aqueous CCA-derived cues (Figure 2E), explained 73% and 91% of the variance, respectively. There were clear differences between the time points, particularly 1–3 hpi compared to 12 hpi with J010-E, and between the two CCA-derived cues. The associated loading plots are available in (Figure S4).

### Mutiplex RT-qPCR Assay 2; other putative settlement/metamorphosis-related genes

Similar to Assay 1, expression levels of 16 GOI changed significantly after 1 hpi with at least one of the cues (*Septin-7*, *LRRC59*, *cAMP/ATF2*, *cAMP/ATF4*, *GABA/GAT-2*, *GABA/GAT-3*, *NADH-ubiq*, *Lectin/nemgalR1*, *Pha-C3*, *amilGFP*, *amilRFP*, *amilCP*, *Amgalaxin-like-1*, *SCRiP3*, *Actin* and *Integrin-alpha1*; Figure 1). The fluorescent protein genes (*amilGFP*, *amilRFP* and *amilCP*) were significantly up-regulated in response to the CCA-derived cues 1 hpi, and all FP genes were significantly down-regulated in response to J010-E but only after 2, 3 and/or 12 hpi (Figure 1). Some genes presented significant differences only after 2 and/or 3 hpi with J010-E (*Septin-1*, *Septin-7*, *LRRC59*, *cAMP/ATF2*, *cAMP/ATF6*, *GABA/ine*, *Lectin/nemgalR1*, *Lectin/nemgalB1*, *amilGFP*, *amilCFP*, *amilRFP*, *Colorless GFP-like* and *SCRiP2*; Figure 1), while others presented constant significant differences at all three sampling time points (*GABA/GAT-2*, *Pha-C3*, Am*galaxin-like-1* and *SCRIP3*; Figure 1). Two genes (*cAMP/ATF4* and *amilGFP*) were consistently up-regulated following exposure to the two CCA-derived cues. Four genes (*Septin-7*, *cAMP/ATF2*, *Lectin/nemgalR1* and *Integrin-alpha1*; Figure 1) were significantly up-regulated after exposure to only HO_org,_ while six genes (*LRRC59*, *NADH-ubiq*, *amilRFP*, *amilCP*, *Amgalaxin-like-1* and *Actin*) were significantly up-regulated following exposure to HO_aq_. As for Assay 1, the PCA score plot (Figure 2B) explained 88% of the variance, and again revealed clear differences in gene regulation following exposure to J010-E or the CCA-derived cues. Additional PCA of the J010-E treatments only (Figure 2D) and of the CCA-derived cues only (Figure 2F) explained 84% and 70% of the variance, respectively. Again the PCA showed clear differences between the early time points (1–3 hpi) and 12 hpi [12], and some separation between the two CCA-derived cues. The associated loading plots are available in (Figure S5).

## Discussion

Biofilms of *Pseudoalteromonas* strain J010, an extract of this bacterium (J010-E) and the purified bacterial metamorphic metabolite, tetrabromopyrrole (TBP), all induce metamorphosis of *A. millepora* larvae without attachment [4]. In contrast, the CCA-derived cues HO_org_ and HO_aq_, repeatedly extracted from *H. onkodes* over three consecutive years (2010–2012), reproducibly induced complete settlement. Substantial changes in expression profiles of 32 genes of interest (GOI; Figures 1 and 2) hypothesized to be involved in larval settlement were observed following one hour post incubation (hpi) with each of these cues (J010-E, HO_org_ or HO_aq_) and prior to any visible signs of morphogenesis. The PCA score plots (Figure 2) show a clear separation between the different cues and time of exposure, thus correlating the early response of genes with downstream biological changes leading to metamorphosis or settlement. Statistical separation of the J010-E treatments from the CCA-derived cues HO_org_ and HO_aq_ was largely explained by the regulation of the immunity-related genes *MAPK-p38*, *AP-1/cJun*, the lectins *millectin*, *HL-2*, tissue remodelling genes *Apextrin*, *Tx60-A*, cysteine-rich protein genes *SCRiP3*, *Amgalaxin-like-1* and *Pha-C3* (Figures 2A–B, S4A and S5A). It should be noted that genes with a low expression difference as compared to the control, even though significant, will most likely have lower biological importance at that early time point in larval development.

*MAPK-p38* and *AP-1/cJun* form the AP-1 early response transcription factor of the Toll/TLR pathway [20] and are associated with the activating transcription factors *cAMP-ATF*. All three cues showed a similar trend in the regulation of *MEKK-1*, *ERK-2*, *MAPK-p38*, *AP-1/cFos*, and *cAMP/ATF2* (Figure 1), indicating common activation of the immunity response in the early developmental (1–3 hpi) phase [5], [6], [8], [10]–[12], [21], [22]. However, *AP-1/cJun* exhibited an opposite response to J010-E over time but showed the same response at 12 hpi and with the two CCA-derived cues. *TRAM* and *NF-κB* (Figure 1) were also differently expressed, with *TRAM* responding to both CCA-derived cues and *NF-κB* responding to J010-E, suggesting different roles during the early stages of settlement and metamorphosis.

Changes in swimming and searching behaviour followed by larval settlement [1], [6] involve cell-surface recognition mechanisms mediated by lectins [23] including substrate selection [8], activation of tissue remodelling [5], [6], [24], nematocyst development [5] and establishment of symbiosis [5]. Initial gene expression patterns in larvae exposed to the CCA-derived cues were characterised by down-regulation of the lectins *millectin*, *CTL-1*, *CTL-2*, *HL-2* and *HL-3* (Figure 1). Exposure to J010-E, however, resulted mostly in up-regulation (1–3 hpi) with gene expression returning to baseline levels after metamorphosis (12 hpi) highlighting opposing roles of these genes in the early stages of metamorphosis (1–3 hpi) and settlement.

Strong up-regulation of *Apextrin* and *Tx60-A* during larval development correlates with tissue remodelling function [5], [7], [8], [10], [25]. Exposure to both CCA-derived cues 1 hpi resulted in down-regulation of both genes, indicating no initiation of tissue re-modelling. Exposure to J010-E elicited the opposite response suggesting tissue remodelling in preparation for metamorphosis had commenced. After 12 hpi with J010-E down-regulation of *Apextrin* was observed correlating with complete metamorphosis without attachment [12].

Galaxin-related proteins are important structural proteins in the calcifying organic matrix of scleractinian corals [26]. *Amgalaxin-like-1* is expressed strongly in larvae and settled primary polyps but not in adult colonies [11]. The up-regulation of *Amgalaxin-like-1* in larvae exposed to J010-E and HO_aq_ (1 hpi to 12 hpi) confirms its involvement in early development. The expression of genes encoding for other cysteine-rich proteins, the SCRiPs proteins, also changed throughout the developmental transition suggesting distinct roles in coral development [22] and bio-mineralization [22]. *SCRiP3* was up-regulated following 1-12 hpi with J010-E, while *SCRiP2* was down-regulated in response to J010-E, similar to that reported previously [6]-[8], [22] highlighting a role in metamorphosis. *Pha-C3*, known to mediate larval development and settlement in *Hydroides elegans* [27] was up-regulated after exposure to J010-E. The expression levels of *SCRiPs*, *Amgalaxin-like-1* and *Pha-*C3 genes did not return to baseline levels, even after metamorphosis in the water column, but continued to be regulated 12 hpi with J010-E [12] correlating with continued development of septa. No change in *Pha-*C3 or SCRiPs expression profiles was observed after short term (1 hpi) exposure to either of the CCA-derived cues.

Although *Actin* did not influence significantly the separation between J010-E and the CCA-derived cues, long-term (12 hpi) exposure to J010-E resulted in its down-regulation coinciding with the formation of non-swimming floating metamorphosed larvae [12], similar to that observed by Hayward et al. [7] while, up-regulation occurred in larvae exposed to HO_aq_ (1 hpi). Furthermore, the chemical composition of the two CCA-derived cues elicited different gene expression profiles 1 hpi even though both cues ultimately resulted in normal settlement 16 hpi. These results provide further evidence that up-regulation of *Actin*, which is linked to movement in coral larvae, likely drives larval settlement.

Differential gene expression of swimming larvae exposed to J010-E over time (1–3 hpi and 12 hpi) enabled further decoupling of GOI involved in different stages of metamorphosis. In both assays, the PCA score plots associated with J010-E only (Figure 2C–D), showed that the early time points were clearly distinct from the 12 hpi time point and correlated with swimming and metamorphosed larva, respectively. This separation was strongly influenced by the regulation of several genes involved in the Toll/TLR pathway, cell recognition, cell adhesion, neurotransmission, FP-related metabolism and calcium handling: *AP-1/cJun*, *MEKK-1*, *Apextrin*, *HL-2*, *GABA/GAT-3*, *Amgalaxin-like-1*, *SCRiP2*, *SCRiP3*, with a lesser influence by *TIR-1*, *TRAF-6*, *AP-1/cFos*, *millectin*, *CTL-2*, *Lectin/nemgalR1*, *Lectin/nemgalB1*, *amilGFP* and *amilCFP* (Figures 2C–D, S4B and S5B). This shift in gene expression profiles suggests that their putative roles may change as the larvae shift from the early (1–3 hpi) to later stage (12 hpi) of coral morphogenesis. Furthermore, the 1 hpi treatments were clearly distinct from those after 2–3 hpi, indicating that genes are being regulated earlier than previously reported [6], thus further supporting a progression through time with respect to gene expression (Figure 2C–D).

In both assays, principal component analysis and the PCA score plots associated with HO_org_ and HO_aq_ (Figure 2E–F) showed a clear distinction between the two treatments, even though both ultimately resulted in settlement. While the expression profiles of 12 GOI (mostly immunity-related genes, Figure 1) were found to be similar following exposure to the two chemically distinct CCA-derived cues, the expression profiles of 15 GOI (Figure 1) were different, that is they were regulated following exposure to only one of the CCA-derived cues, but were never oppositely regulated. The separation of the two treatments was influenced strongly by the regulation of *TIR-1*, *AP-1/cJun*, *AP-1/cFos*, *MEKK-1*, *ERK-2*, *Colorless GFP-like*, *amilGFP* and *cAMP/ATF6*, with a lesser influence by *NF-κb*, *MAPK-p38*, *C3*, *Apextrin*, *CTL-2* and *Tx60-A* (Figures 2E–F, S4C and S5C). The immunity-related Toll/TLR pathway genes contributed mostly to the separation of these treatments. These observations raise the possibility that 1) more than one component influenced/regulated the immunity-related processes involved in both attachment and metamorphosis, or 2) that a synergistic effect was causative for this observation. Since the cnidarian gene cascade leading to settlement has not yet been fully elucidated [10], it cannot be ruled out that numerous pathways may be regulated by many different components within the environment (i.e. bacterial biofilm, CCA).

Regulation of the fluorescent protein (FP) genes influenced separation between the different time points for J010-E exposure and also between the two settlement cues. While no initial significant differences were observed 1 hpi with J010-E, there was a downward trend in expression levels of *amilGFP*, *amilCFP* and *Colourless GFP-like* (Figure 1), with all FP genes down-regulated after 12 hpi [12]. FP genes (particularly RFP and CP) were reportedly up-regulated in compromised corals, activating an antioxidant response [28] and given the strong down-regulation of FP genes following exposure to J010-E it is likely the larvae are responding to TBP [29] in J010-E. FP genes *amilGFP*, *amilRFP* and *amilCP* (Figure 1) were mostly up-regulated 1 hpi with HO_aq_ indicating a different role in the early stages of settlement compared to metamorphosis. Similarly, Beltran-Ramirez [30] reported a strong green FP (GFP) signal in swimming larvae, up-regulation of FP in both the ectoderm and endoderm of *A. millepora* larvae undergoing settlement and down-regulation in ectodermal tissue after metamorphosis [30]. The current study corroborates this, providing further evidence that FP genes are differentially regulated in swimming larvae post exposure to metamorphosis- or settlement-inducing cues and prior to visible metamorphic changes. Up-regulation of *amilCP* 1 hpi with HO_aq_ further supports an earlier observation that chromoprotein (CP) gene expression was detected primarily during gastrulation and was mostly endodermal [30]. Furthermore, *A. tenuis* larvae exposed to CCA chips showed an increase in red FP (RFP) expression prior to but not after settlement, while cyan FP (CFP) expression increased post settlement [21]. Exposure of *A. millepora* larvae to J010-E resulted in down-regulation of these two FP genes 2-12 hpi which correlated to complete metamorphosis.

The CCA-derived cue HO_org_ represents the organic-soluble, hydrophobic, low molecular weight components of the *H. onkodes* extract while HO_aq_ was derived from an autoclaved aqueous extract of CCA and contained high molecular weight compounds. Both CCA-derived cues ultimately induced complete settlement, producing competent polyps that were able to acquire *Symbiodinium* and deposit a skeleton (data not shown). High specificity of large molecule-mediated recognition has previously been observed in a wide variety of biological systems [31]. Based on these observations HO_aq_ may be acting similarly to lectins at the cell surface, activating the downstream MAPK pathway, whereas HO_org_ influences, possibly within the cell, changes in searching behaviour. It should also be noted that like any other surface, CCAs are known to harbour bacterial biofilms [32] and that these may indeed be the source of the observed inductive capacity of CCA fractions on coral larval settlement. Our findings show that two different and distinct chemical classes can elicit the same settlement response by regulating a different set of genes, highlighting that there is more than one pathway involved.

This study has enabled further elucidation of gene function(s) through a time series investigation into their responses to CCA- and bacteria-derived cues. Importantly, this gene regulation differed not only with regard to the time of exposure but also to the morphogenetic outcome; settlement (CCA-derived cues) or metamorphosis without attachment (bacteria-derived cue). It should be noted that while the genes investigated in this study are known for *A. millepora*, the gene regulation pathways have not been fully elucidated and are mostly based on vertebrate systems, providing only putative functionality. Even so, the results of this study support the notion that regulation of specific genes is driven by environmental cues throughout the different phases of *A. millepora* larval settlement, from initial induction to cellular differentiation and complete metamorphosis resulting in settlement.

## Supporting Information

Figure S1 **Differential gene expression following exposure to bacteria- and CCA-derived cues.** Significant (*p<0.05*) change in gene regulation is given as the % difference compared to the control. Dark and light green (+) represents those genes that were up-regulated (X>20, 20≤X<0); orange and red (−) represents those that were down-regulated (0>X≥−20, X<−20). Data normalised to the three most stable genes. Assay 1 CCA: *Rps7*, *ATF4/5* and *NF-kB*, Assay 2 CCA: *Rps7*, *Amgalaxin-like-1* and *Lectin/nemgalB2*, Assay 1 J010-E: *Rpl9*, *CTL-2* and *ATF4/5*; Assay 2 J010-E: *amilRFP*, *Rps7* and *cAMP/ATF4*. Data from the 12 hpi experiment (high concentration treatment and complete metamorphosis) was taken from Siboni et al. [12], which also includes full protein names and description of the genes.
(TIF)

Figure S2 **Differential gene expression following exposure to CCA-derived cues, HO_org_ and HO_aq_.** The boxplots represent change in gene regulation as % difference from control. Only cases which present significant (*p<0.05*, Kruskal–Wallis followed by multiple comparisons of mean ranks) differences from control were included. For each gene, the left region represents HO_org_ and the right region represents HO_aq_.
(TIF)

Figure S3 **Differential gene expression following exposure to the bacterial cue J010-E over time (1–3 hpi).** The boxplots represent changes in gene regulation expressed as the % difference from the control. Only cases that present significant (*p<0.05*, Kruskal–Wallis followed by multiple comparisons of mean ranks) differences from the control were included. For each gene, the left region represents 1 hpi, the middle region represents 2 hpi and the right region represents 3 hpi. 12 hpi data is available in Siboni et al. [12].
(TIF)

Figure S4 **PCA loading plots of Assay 1 following exposure to settlement and metamorphosis cues.** PCA loading plots (PC1 and PC2) of the % difference in gene expression compared to the control for A) J010-E 1–12 hpi and HO_org/aq_, B) J010-E 1–12 hpi and C) HO_org/aq_.
(TIF)

Figure S5 **PCA loading plots of Assay 2 following exposure to settlement and metamorphosis cues.** PCA loading plots (PC1 and PC2) of the percentage difference in gene expression compared to the control for A) J010-E 1–12 hpi and HO_org/aq_. B) J010-E 1–12 hpi and C) HO_org/aq_.
(TIF)
