# Supplementary figures and images for: Gene Expression Patterns during the Early Stages of Chemically Induced Larval Metamorphosis and Settlement of the Coral Acropora millepora
Source: PLoS One. 2014 Mar 14;9(3):e91082. doi: 10.1371/journal.pone.0091082 (PMC3954620; doi:10.1371/journal.pone.0091082)

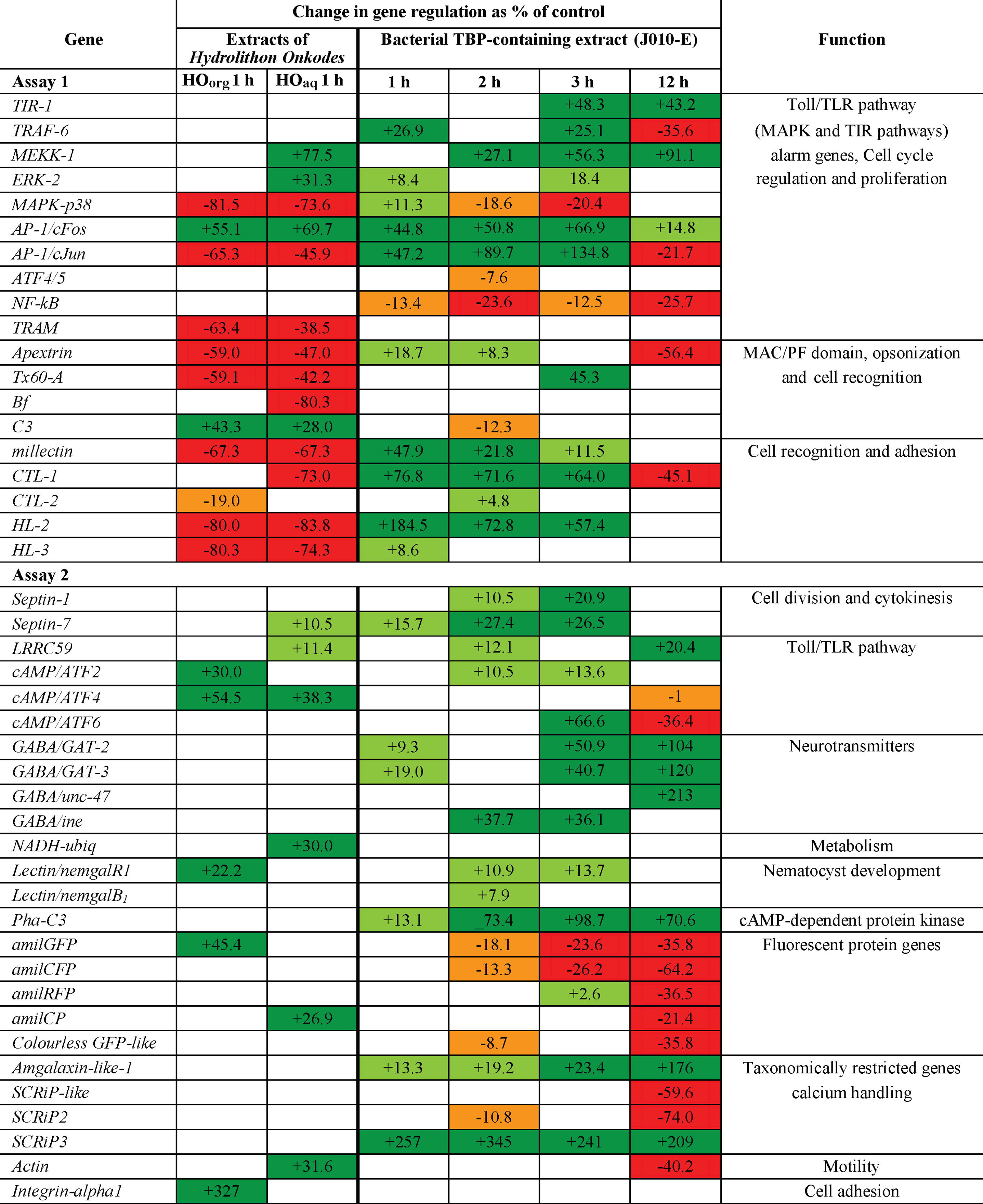

Supplement: Figure S1 — Differential gene expression following exposure to bacteria- and CCA-derived cues. Significant (p<0.05) change in gene regulation is given as the % difference compared to the control. Dark and light green (+) represents those genes that were up-regulated (X>20, 20≤X<0); orange and red (−) represents those that were down-regulated (0>X≥−20, X<−20). Data normalised to the three most stable genes. Assay 1 CCA: Rps7, ATF4/5 and NF-kB, Assay 2 CCA: Rps7, Amgalaxin-like-1 and Lectin/nemgalB2, Assay 1 J010-E: Rpl9, CTL-2 and ATF4/5; Assay 2 J010-E: amilRFP, Rps7 and cAMP/ATF4. Data from the 12 hpi experiment (high concentration treatment and complete metamorphosis) was taken from Siboni et al. [12], which also includes full protein names and description of the genes. (TIF) [file pone.0091082.s001.tif]

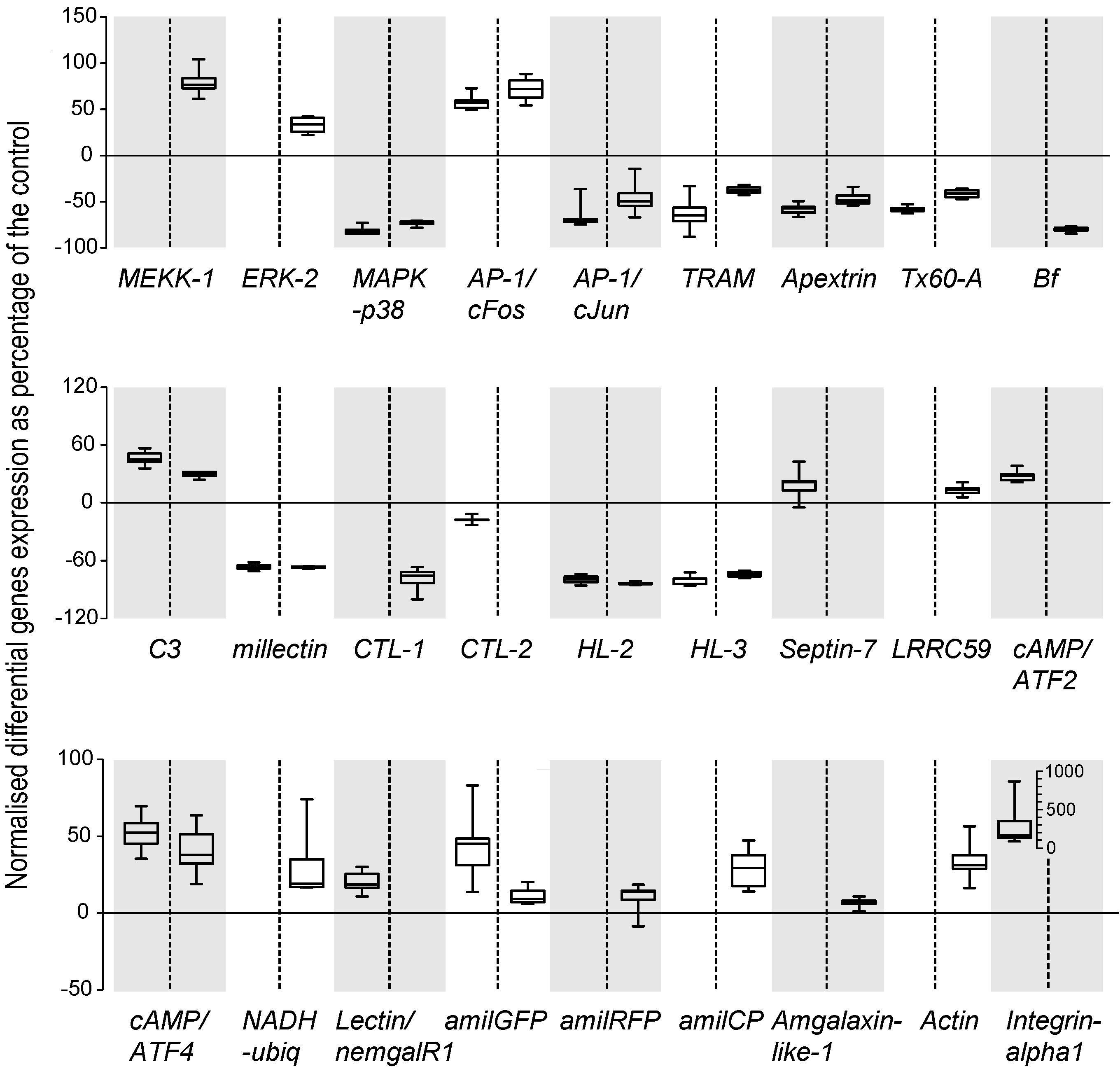

Supplement: Figure S2 — Differential gene expression following exposure to CCA-derived cues, HOorg and HOaq. The boxplots represent change in gene regulation as % difference from control. Only cases which present significant (p<0.05, Kruskal–Wallis followed by multiple comparisons of mean ranks) differences from control were included. For each gene, the left region represents HOorg and the right region represents HOaq. (TIF) [file pone.0091082.s002.tif]

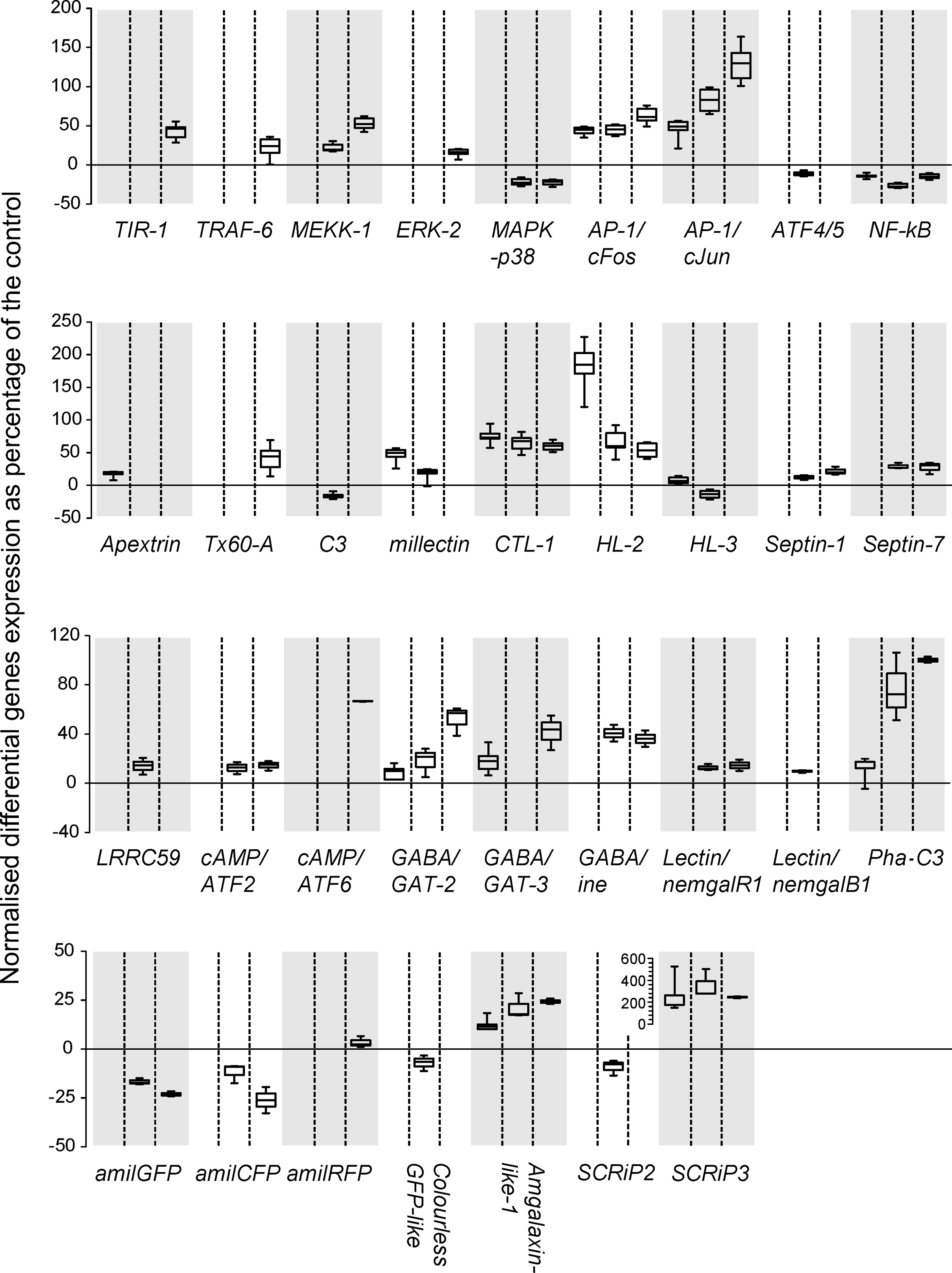

Supplement: Figure S3 — Differential gene expression following exposure to the bacterial cue J010-E over time (1–3 hpi). The boxplots represent changes in gene regulation expressed as the % difference from the control. Only cases that present significant (p<0.05, Kruskal–Wallis followed by multiple comparisons of mean ranks) differences from the control were included. For each gene, the left region represents 1 hpi, the middle region represents 2 hpi and the right region represents 3 hpi. 12 hpi data is available in Siboni et al. [12]. (TIF) [file pone.0091082.s003.tif]

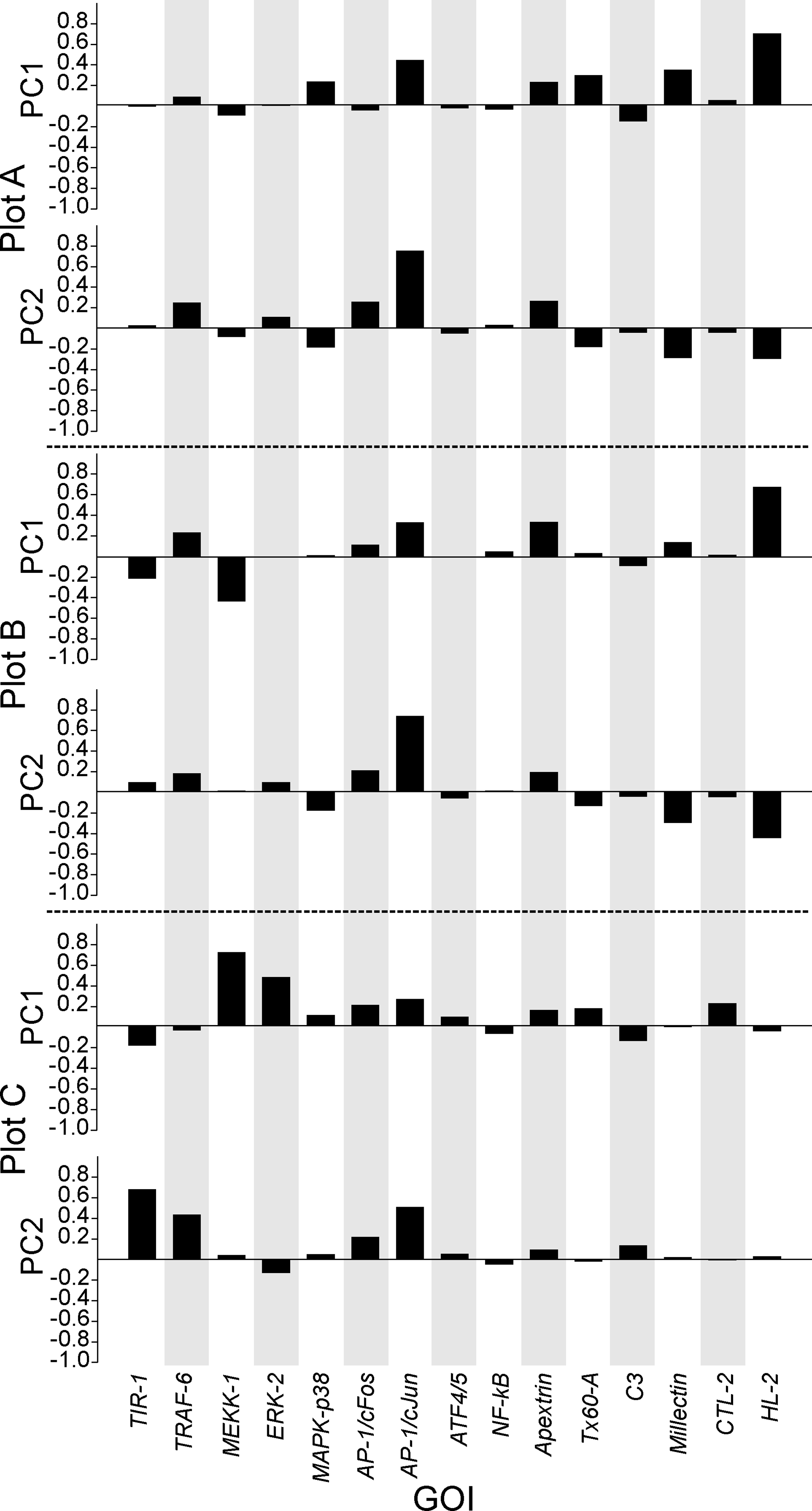

Supplement: Figure S4 — PCA loading plots of Assay 1 following exposure to settlement and metamorphosis cues. PCA loading plots (PC1 and PC2) of the % difference in gene expression compared to the control for A) J010-E 1–12 hpi and HOorg/aq, B) J010-E 1–12 hpi and C) HOorg/aq. (TIF) [file pone.0091082.s004.tif]

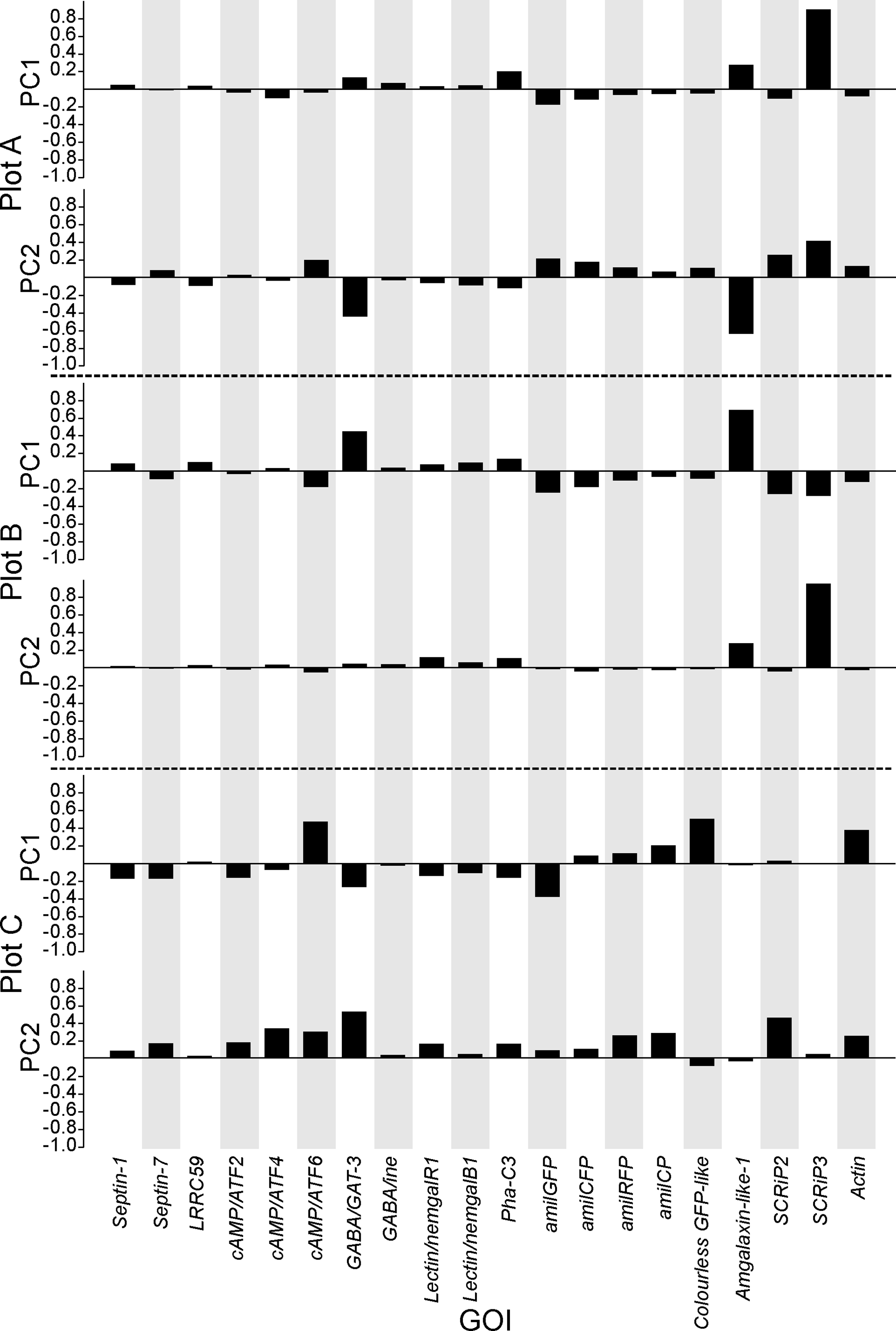

Supplement: Figure S5 — PCA loading plots of Assay 2 following exposure to settlement and metamorphosis cues. PCA loading plots (PC1 and PC2) of the percentage difference in gene expression compared to the control for A) J010-E 1–12 hpi and HOorg/aq. B) J010-E 1–12 hpi and C) HOorg/aq. (TIF) [file pone.0091082.s005.tif]
